# Supplementary material for: Unexpected large eruptions from buoyant magma bodies within viscoelastic crust
Source: Nat Commun. 2020 May 15;11:2403. doi: 10.1038/s41467-020-16054-6 (PMC7229005; doi:10.1038/s41467-020-16054-6)
Supplement: Supplementary file 1 — Supplementary Information [file 41467_2020_16054_MOESM1_ESM.pdf]

Unexpected large eruptions from buoyant magma bodies within viscoelastic crust

Sigmundsson et al.

Supplementary Information

Supplementary Note 1

Supplementary References

Supplementary Figures

Supplementary Note 1

Bárðarbunga. On 16 August 2014, intense seismicity started in the vicinity of Bárðarbunga caldera. During the first 24 hours, a cluster of earthquakes initially propagated to the southeast of the caldera then towards the northeast for the next 2 weeks. Seismicity propagated ~ 40 km beneath the ice-cap, then continuing another 8 km outside the glacier to an eruption site at the Holuhraun plain. This intense seismicity (over 22,000 earthquakes) and associated deformation was representative of a segmented lateral dike injection[1, 2]. A minor effusive eruption started at midnight on 29 August at Holuhraun, lasting only a few hours. The main eruption began on 31 August by opening a 1.8 km long fissure segment issuing spectacular lava fountains with heights reaching 70 m (ref. 3). This fissure remained active throughout the entire eruption, which ended on the 27 February 2015. During this six-month

period the eruption produced a lava field with a volume of 1.4 ± 0.2 km^3. The eruption was accompanied by the gradual collapse of Bárðarbunga caldera of up to 65 m (ref. 4) and intense seismicity on caldera bounding faults with 77 M>5 earthquakes.

A combination of geodetic observations (InSAR and cGPS), radar profiling over the Bárðarbunga caldera, seismic and petrological analysis indicated that the observed deformation and seismicity was the result of melt withdrawal from a magma body beneath the Bárðarbunga caldera, slip on caldera ring faults and the transfer of magma along a 48 km long dike which was feeding the major effusive eruption on the Holuhraun plain. Magma that drained from beneath the caldera was initially emplaced in a dike, and then erupted at the far end of the dike. Good GPS and InSAR coverage of the events allowed detailed modelling. The calculated volume change at the end of the eruption, associated with magma withdrawal from the modelled sill ~10 km deep beneath Bárðarbunga, was -1.9 ± 0.1 km^3 and 0.7 ± 0.04 km^3 for the dike volume[5]. The dike opening mostly occurred over a period of weeks, whereas the caldera subsidence at Bárðarbunga occurred gradually over a period of six months, simultaneous with the six-month long lava flow eruption at the far end of the dike.

The 2014-2015 events followed a decade of growing seismicity at Bárðarbunga. A step-up in the number of small earthquakes occurred in mid-May 2014, causing a marked increase in the rate of seismic moment release. Approximately 800 earthquakes of magnitudes up to M3.7 were recorded after 1 May and until 16 August, inside and around the caldera. At about the same time, the nearest continuous GPS station, installed in the fall of 2013, at site VONC, began to move at a faster rate away from the caldera (Fig. 1 and Supplementary Fig. 1). The seismicity and deformation observations suggest renewed magma inflow into the roots of Bárðarbunga. However, the recorded deformation signal associated with this deformation anomaly is only about 12 mm. The spatial pattern of seismicity was coherent with and in the same location as the activity of the previous years, within 6 km northeast of the caldera rim.

The increasing earthquake activity observed at Bárðarbunga over the decade preceding 2014 is interpreted to reflect high crustal stress and conditions close to failure during this period, with a small amount of additional magma inflow occurring in the months prior to 16 August, which brought the magma body to failure.

We suggest that prior to the caldera collapse, the caldera faults of Bárðarbunga were weak, following the series of M5+ earthquakes in the 1975-1996 period, and ring faulting and eventual ring dike injection 29-30 September 1996 (refs. 6, 7). Modelling of geodetic data suggest other intrusive events may have occurred in earlier decades influencing the present stress field[8]. After the failure of the magma body under Bárðarbunga on 16 August 2014, we suggest magma initially flowed vertically upward prior to being captured by the deviatoric stress field in the brittle crust, governed by topography and regional tectonics (and forming the laterally propagating dike). Accordingly, the magma would have utilized a zone of weakness in the southeastern part of the caldera boundary. This is where the 2014 laterally propagating dike originated[1, 9] and the probable source location of the 1996 dike[6, 7]. Furthermore, ice cauldrons were formed outside the Bárðarbunga caldera in 2014 (Fig. 1), suggesting heat was delivered to the base of the ice cap[1]. Following the 2014-2015 events geothermal activity picked up in a few places along the Bárðarbunga caldera boundary, with the most active area in the southeast part of the caldera. The eastern part of the caldera boundary is much less seismically active than its northern and southern parts (Fig. 1), suggesting it is hot enough for ground displacement and magma flow to occur without producing noticeable earthquake activity.

Supplementary Figures

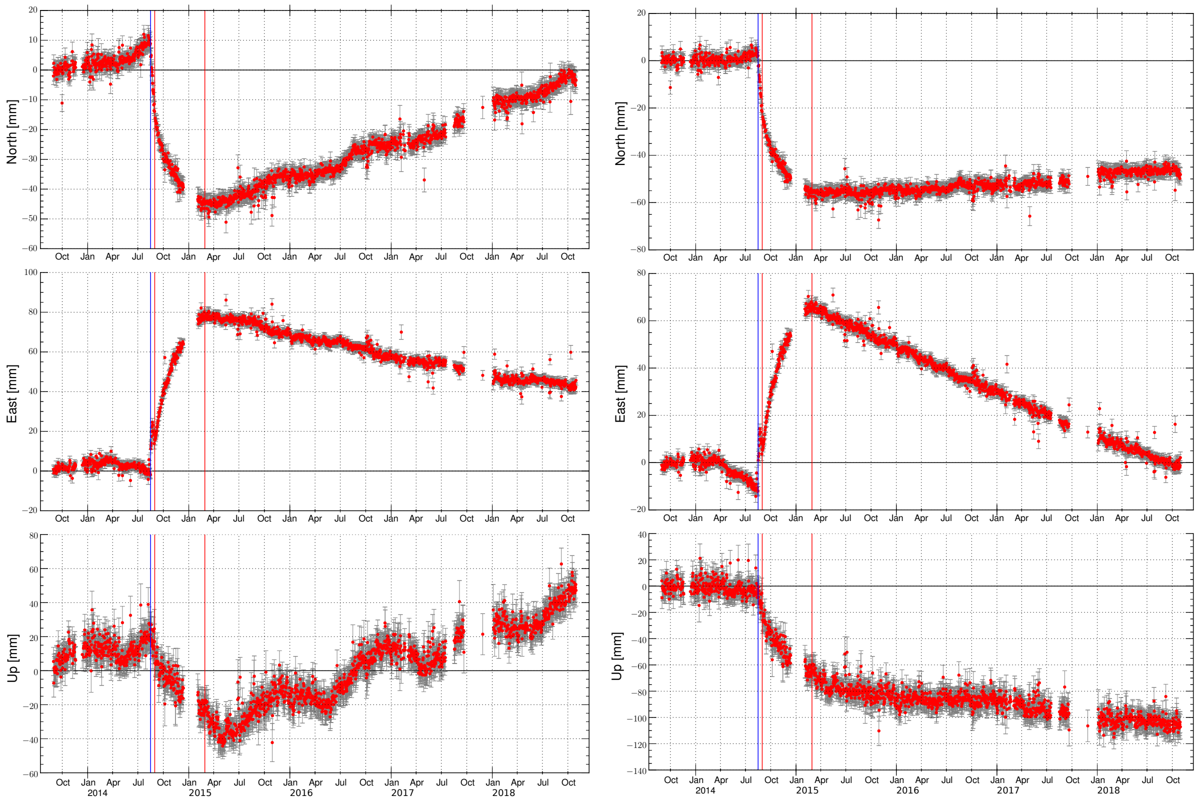

Supplementary Figure 1. Three-dimensional times series of displacement at VONC GPS site. Time series from GPS station VONC in Vonarskarð, west of Bárðarbunga (see Fig. 1 for location). Blue vertical lines show the onset of the Bárðarbunga rifting, red vertical lines mark the Holuhraun eruption period. Left panels show unfiltered time series in stable North American plate reference frame. Right panels show the detrended time series, where annual periodic signal has been subtracted as well as a linear velocity estimated for the period prior to May 2014. The detrended time series for the East component of displacement reveals an inflation signal beginning 3-4 months prior to rifting at Bárðarbunga (horizontal displacement to the west; away from the caldera). At the onset of Bárðarbunga rifting event a superposition of signals related to the formation of a dike extending to Holuhraun and deflation is observed. After the eruption onset, an exponentially decaying deflation signal is observed superimposed on an inflation signal, best witnessed in the East component of displacement.

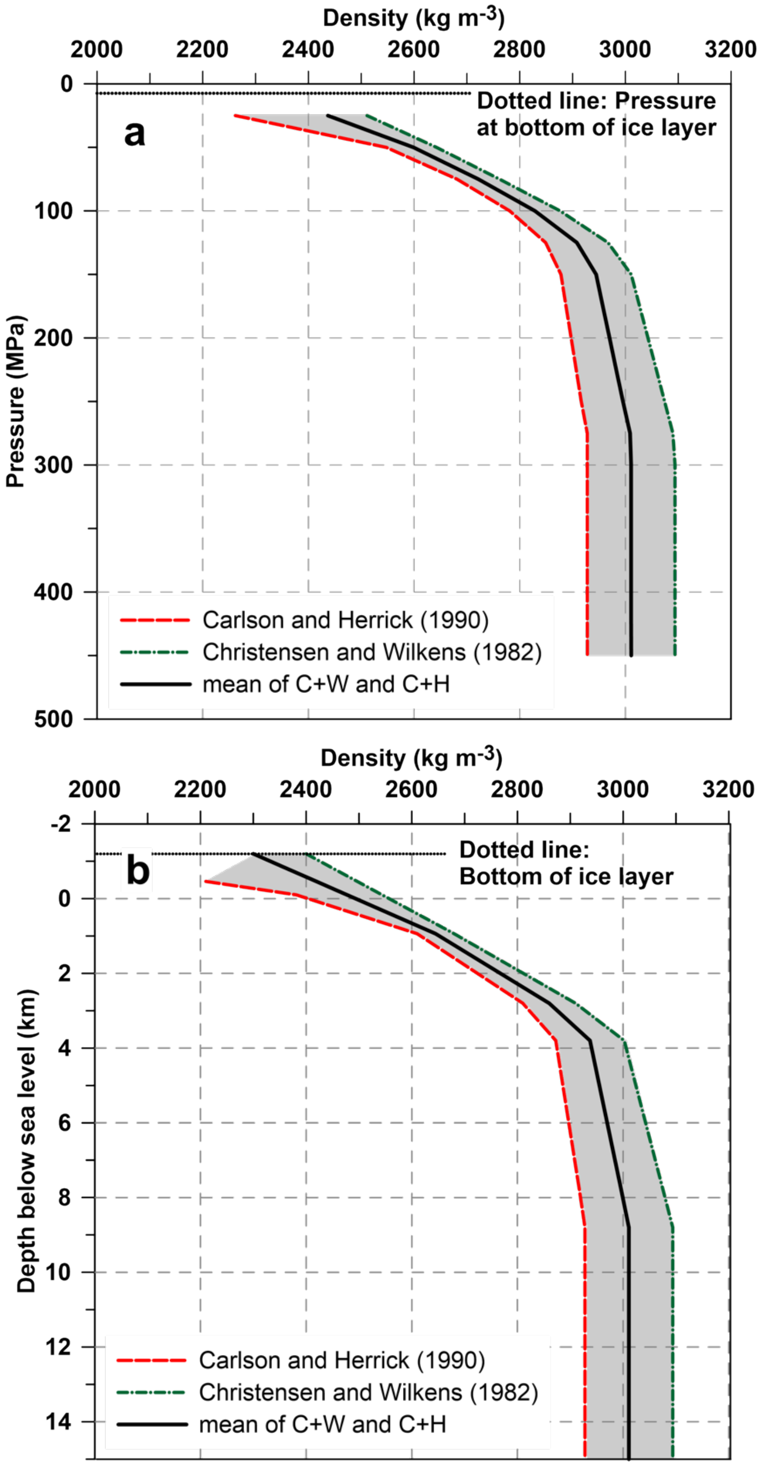

Supplementary Figure 2. Density-lithostatic pressure systematics for Bárðarbunga

Density-pressure (a) and density-depth (b) systematics for Bárðarbunga based on the empirical density models (Methods).

Supplementary References:

1. Sigmundsson, F., *et al*. Segmented lateral dyke growth in a rifting event at Bárðarbunga volcanic system, Iceland. *Nature* **517**, 191–195 (2015).

2. Ágústsdóttir, T., Winder, T., Woods, J., White, R. S., Greenfield, T., Brandsdóttir, B. Intense seismicity during the 2014–15 Bárðarbunga-Holuhraun rifting event, Iceland, reveals the nature of dike-induced earthquakes and caldera collapse mechanisms. *J. Geophys. Res. Solid Earth* **124**, 8331-8357 (2019).

3. Pedersen, G. B. M. *et al*. Lava field evolution and emplacement dynamics of the 2014–2015 basaltic fissure eruption at Holuhraun, Iceland. *J. Volc. Geotherm. Res.* **340**, 155-169 (2017).

4. Gudmundsson, M. T., *et al*. Gradual caldera collapse at Bárdarbunga volcano, Iceland, regulated by lateral magma outflow. *Science* **353**, 6296 (2016).

5. Parks, M. M., et al. Evolution of deformation and stress changes during the caldera collapse and dyking at Bárdarbunga, 2014–2015: Implication for triggering of seismicity at nearby Tungnafellsjökull volcano. *Earth Planet Sci. Lett.* **462**, 212-223 (2017).

6. Einarsson, P. *et al*. Center of the Iceland hotspot experiences volcanic unrest. *Eos* **78**, 374-375 (1997).

7. Pagli, C. *et al*. Crustal deformation associated with the 1996 Gjálp subglacial eruption, Iceland: InSAR studies in affected areas adjacent to the Vatnajökull ice cap. *Earth Planet. Science Letters* **259**, 24-33 (2007).

8. Spaans, K. Hooper, A. Insights into the stress field around Bárðarbunga Volcano from the 2014/2015 Holuhraun rifting event. *J. Geophys. Res. Solid Earth* **123**, 3238–3249 (2018).

9. Heimisson, E. R., Hooper, A., Sigmundsson, F. Forecasting the path of a laterally propagating dike. *J. Geophys. Res. Solid Earth* **120**, 8774-8792 (2015).
